# Molecular Effects of Indocyanine Green-Photodynamic Therapy on Programmed Cell Death Pathways in T98G and U-118MG Glioblastoma Cells—An RT-qPCR Study

**DOI:** 10.3390/cimb48070659

**Published:** 2026-06-26

**Authors:** Klaudia Dynarowicz, Joanna Katarzyna Strzelczyk, Dorota Bartusik-Aebisher, Wiktoria Mytych, Alina Pietryszyn-Bilińska, Aleksandra Kawczyk-Krupka, Dorota Hudy, Oliwia Trzaskoś, Jacek Tabarkiewicz, David Aebisher

**Affiliations:** 1 Department of Biochemistry and General Chemistry, Faculty of Medicine, University of Rzeszów, 35-310 Rzeszów, Poland; kdynarowicz@ur.edu.pl (K.D.);; 2 Department of Medical and Molecular Biology, Faculty of Medical Sciences in Zabrze, Medical University of Silesia in Katowice, Jordana 19 St., 41-808 Zabrze, Poland; 3 English Division Science Club, Faculty of Medicine, University of Rzeszów, 35-310 Rzeszów, Poland; 4 Department of Internal Medicine, Angiology and Physical Medicine, Faculty of Medical Sciences in Zabrze, Medical University of Silesia in Katowice, 15 Stefana Batorego St., 41-902 Bytom, Poland; 5 Department of Internal Diseases, Angiology and Physical Medicine, Center for Laser Diagnostics and Therapy, Medical University of Silesia, Batorego 15, 41-902 Bytom, Poland; 6 Department of Human Immunology, Faculty of Medicine, University of Rzeszów, 35-310 Rzeszów, Polandjtabarkiewicz@ur.edu.pl (J.T.); 7 Department of Photomedicine and Physical Chemistry, Faculty of Medicine, University of Rzeszow, 35-310 Rzeszów, Poland

**Keywords:** glioblastoma, photodynamic therapy, indocyanine green, apoptosis, ferroptosis, gene expression, T98G, U-118MG, RT-qPCR

## Abstract

Glioblastoma multiforme (GBM) remains one of the most aggressive primary brain tumors with poor prognosis despite multimodal therapy. Photodynamic therapy (PDT) using indocyanine green (ICG) is an emerging adjuvant approach aimed at eliminating residual tumor cells after resection. While ICG-PDT exerts cytotoxic effects, its impact on molecular pathways regulating programmed cell death in glioma cells is not fully understood. In this study, T98G and U-118MG glioblastoma cells were divided into four groups: untreated control, light-only (10 min broadband irradiation), ICG-only (15 min incubation), and ICG-PDT (15 min ICG + 10 min broadband irradiation). Relative mRNA expression of apoptosis-related genes (BAX, BCL2, CASP3, FAS) and ferroptosis-related genes (GPX4, ACSL4, SLC7A11, GCH1) was quantified 24 h post-treatment by RT-qPCR using the 2^−ΔΔCt^ method. ICG-PDT significantly reduced cell viability to 67.79% ± 3.39% (vs. 86.66% ± 4.33% in control), confirming effective phototoxicity. No statistically significant differences in mRNA levels were observed for any of the investigated genes across the groups (one-way ANOVA and Kruskal–Wallis, all *p* > 0.05). The largest non-significant deviation was a mild decrease in GPX4 (fold change 0.87) in the ICG-PDT group. Fluctuations in GCH1 were accompanied by high variance, likely reflecting technical noise rather than a true biological trend. The mRNA BAX/BCL2 ratio remained stable (~30) across all conditions. In contrast, the U-118MG line showed greater transcriptional sensitivity, with statistically significant decreases in CASP3 (*p* = 0.012) and ACSL4 (*p* = 0.031) expression, along with downward trends in BCL2 and GPX4 following ICG-PDT. ICG-PDT does not induce significant transcriptional changes in the analyzed genes T98G at the 24 h time point under the applied experimental conditions. In U-118MG cells, moderate transcriptional engagement of both apoptotic and ferroptotic routes was observed. Further studies at the protein and functional levels, across multiple time points and models, are warranted to fully elucidate the mechanisms of ICG-PDT in glioblastoma.

## 1. Introduction

**Figure 1:**
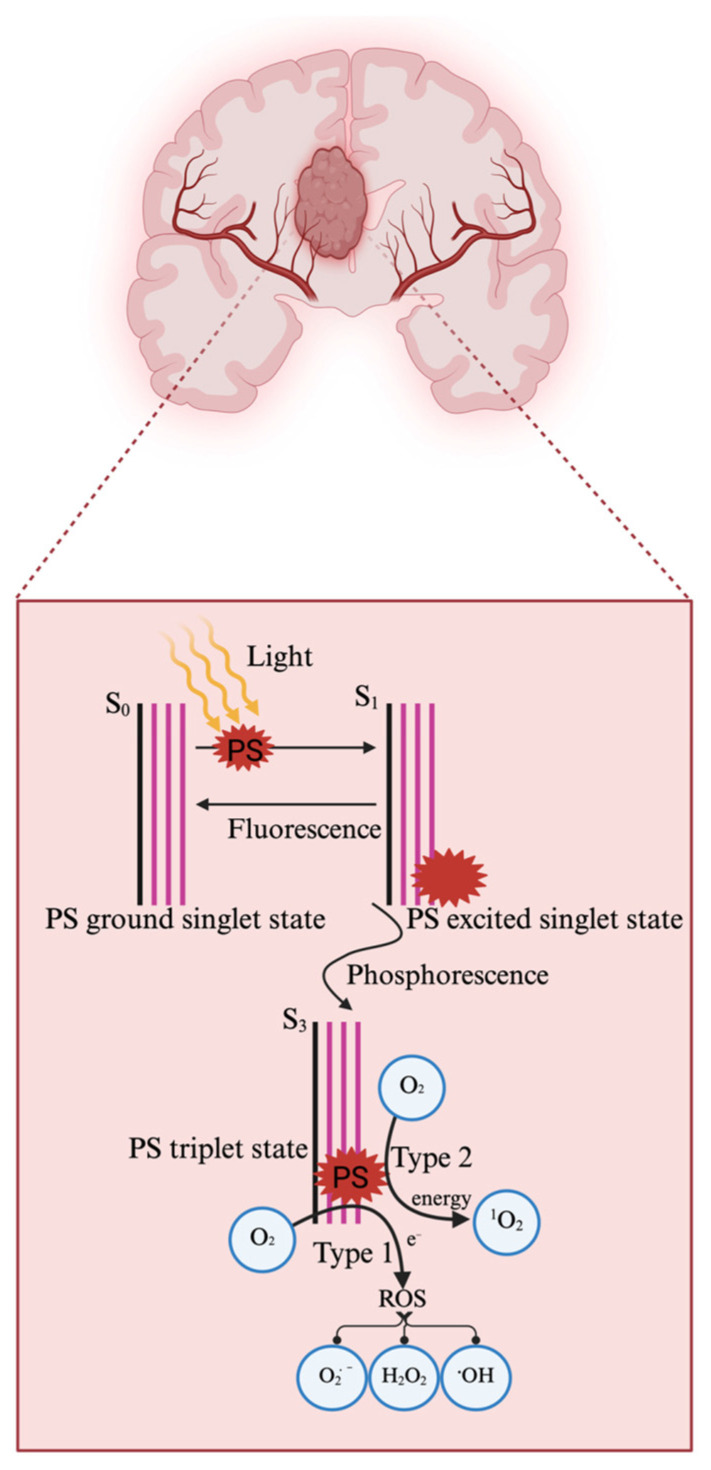
Schematic representation of the PDT mechanism in glioblastoma treatment using a photosensitizer (PS). The upper panel illustrates the clinical context: selective accumulation of the photosensitizer in the glioblastoma tumor mass within the brain, followed by localized light irradiation to activate the therapeutic effect while sparing surrounding healthy tissue. The lower panel depicts a modified Jablonski diagram [20] explaining the photophysical and photochemical processes. Upon absorption of light (yellow wavy arrows), the PS in its ground singlet state (S_0_) is promoted to an excited singlet state (S_1_). From the excited singlet state, the PS can return to the ground state via fluorescence (emitted light) or undergo intersystem crossing (ISC) to the longer-lived triplet excited state (T_1_). In the triplet state, two main photochemical pathways lead to the generation of ROS. Type I reaction (left branch): Electron or hydrogen transfer from the triplet PS to surrounding substrates (e.g., biomolecules) or molecular oxygen (O_2_), producing radical species such as superoxide anion (O_2_^•−^), hydroxyl radicals (•OH), hydrogen peroxide (H_2_O_2_), and other ROS. Type II reaction (right branch, dominant in many PDT systems): Direct energy transfer from the triplet PS to ground-state triplet oxygen (^3^O_2_), generating highly cytotoxic singlet oxygen (^1^O_2_). Both pathways result in oxidative damage to cellular components (lipids, proteins, nucleic acids), leading to mitochondrial dysfunction, membrane peroxidation, apoptosis, ferroptosis, or other forms of programmed cell death in tumor cells. Fluorescence and phosphorescence pathways are indicated as non-productive returns to the ground state. The scheme emphasizes the oxygen-dependent nature of PDT and the central role of ROS (primarily ^1^O_2_ in Type II) in mediating cytotoxicity.

Gliomas are a group of primary tumors of the central nervous system (CNS), originating from glial cells (astrocytes, oligodendrocytes, or ependymocytes) [1]. They constitute the most common primary brain tumors in adults, accounting for approximately 30–40% of all primary CNS tumors, and are characterized by high malignancy and infiltrative growth, which prevents complete surgical removal [2]. According to the current WHO classification, gliomas are divided into grades I–IV. Glioblastoma multiforme is the most aggressive, with a median survival of only 12–15 months despite multimodal treatment [3]. The main reasons for this poor prognosis include the blood–brain barrier (BBB), which limits drug access, the tumor’s molecular heterogeneity, the presence of cancer stem cells, and the rapid development of resistance to chemotherapy (temozolomide) and radiotherapy [4]. Standard treatment includes maximally safe tumor resection, radiation therapy, and alkylating agent chemotherapy. Despite the continuous development of neurosurgical techniques, the rate of complete resection remains low, and recurrence occurs in over 90% of patients within 6–9 months [5]. Therefore, for many years, there has been a search for adjuvant therapies that are highly selective for cancer cells while preserving healthy brain tissue. One of the most promising methods in the treatment of gliomas is photodynamic therapy (PDT) (Figure 1). This two-stage treatment modality is based on the interaction of three individually harmless components: a photosensitizer, light of the appropriate wavelength, and molecular oxygen [6]. The photosensitizer is administered intravenously or locally and accumulates in tumor tissue primarily through the enhanced permeability and retention (EPR) effect and binding to plasma proteins. After the accumulation period, the tumor area is irradiated with laser or LED light [7]. In the presence of oxygen, ICG can undergo both Type I and Type II photochemical reactions, although its singlet oxygen quantum yield is relatively low compared to traditional photosensitizers. When excited in the near-infrared range, the photothermal effect may also significantly contribute to cytotoxicity, especially due to ICG’s tendency to form aggregates. Under the hypoxic conditions characteristic of glioblastoma, Type I reactions (producing radical species such as superoxide anion and hydroxyl radicals) may become more prominent. ROS generated by these processes cause direct damage to mitochondria, cell membranes, cytoskeletal proteins, and DNA, while simultaneously activating various cell death pathways including apoptosis, autophagy, and potentially ferroptosis [8,9]. Additional effects of PDT include the destruction of tumor microcirculation and stimulation of an anti-tumor immune response [10]. It is important to note that, unlike 5-ALA-derived protoporphyrin IX, ICG primarily acts as a vascular and interstitial photosensitizer with a lower efficiency in intracellular singlet oxygen generation. In neuro-oncology, PDT is primarily used intraoperatively, following macroscopic tumor resection, to destroy microscopic tumor remnants in the postoperative bed [11]. This method has already been registered in several countries (including Japan–sodium porfimer; Europe–clinical trials with 5-ALA), and the results of Phase II/III studies indicate prolonged progression-free survival and a reduced risk of early relapse [12]. The advantages of PDT in gliomas include high selectivity, lack of cumulative toxicity, the possibility of repeat treatment, and synergy with other modalities [13]. To better understand the molecular mechanisms of PDT action, glioma cell resistance, and potential targets for combination therapy, transcriptome studies (RNA-seq, microarrays) and gene expression profiling (RT-qPCR) before and after radiation are increasingly being conducted [14,15]. Such analyses allow the identification of key signaling pathways (e.g., *NF-κB*, *Nrf2*, *HIF-1α*, the *BCL-2/BAX* apoptotic pathway), genes associated with antioxidant defense, and markers of PDT resistance [16,17,18]. The results of these studies provide a basis for designing combination therapies (PDT + inhibitors of selected pathways) and pave the way for precision medicine in the treatment of gliomas [19].

The *BAX* gene encodes a pro-apoptotic protein that belongs to the BCL-2 family and is a major factor in controlling programmed cell death as well as the induction of mitochondrial membrane permeabilization and the release of cytochrome c to cause caspase activation and apoptosis [21]. *BAX* is inactivated or downregulated in numerous types of cancers, and this condition helps cancer cells resist apoptosis, thus supporting tumor growth [22]. Research shows that a decrease in BAX levels is associated with an increase in malignancy, including glioblastoma in gliomas, and with a low clinical prognosis following surgery, radiation and chemotherapy [23]. Furthermore, recent studies highlight that the severe hypoxic microenvironment typical of glioblastoma dynamically modulates BCL-2 family proteins, including BAX, altering mitochondrial apoptotic thresholds. This hypoxia-driven regulation promotes a highly invasive and chemoresistant phenotype, suggesting that targeting the BAX pathway in combination with hypoxia-reversing therapies or metabolic modulators may yield new therapeutic avenues for resistant tumors [24,25].

The primary antiapoptotic of the BCL-2 family is BCL-2, which prevents programmed cell death due to its ability to block mitochondrial membrane permeabilization, and release of cytochrome c and caspase activation, which results in cell survival [26]. BCL-2 is frequently overexpressed in glioma, particularly glioblastoma multiforme (GBM, WHO grade IV), and can have an antiapoptotic instead of a proapoptotic effect, which facilitates resistance to apoptosis, radiotherapy, and chemotherapy (including temozolomide) and tumor progression and recurrence following therapy [27]. Clinical and experimental studies have indicated that the rise in BCL-2 expression is associated with the increase in the grade of glioma, the invasion and migration of tumor cells, and the poor outcome in the patient following his or her resection and conventional therapy [28].

Caspase-3 (CASP3) is among the predominant agents of the apoptotic cleavage and proteolytic degradation of numerous cellular targets, such as DNA enzyme, DNase inhibitors that produces cell damage and cell death that cannot be reversed [29]. The role of CASP3 is rather paradoxical in gliomas, and more so in GBM on the one hand, to induce apoptosis, radiotherapy, or many experimental drugs, the activation of caspase-3 is imperative, whereas on the other hand, the artificial activation about caspase-3 in its turn correlates with worse prognosis in gliomas, worse patient survival, when combined with an IDH-wildtype mutation, through atypical, non-apoptotic activity, as pro-angiogenic activity in the post-irradiation microenvironment, repopulation of tumor cells by dying cells, and enhanced glioma migration and invasiveness [30,31]. In GBM tissue, immunohistochemical activation of cleaved CASP3 is often less than that of antiapoptotic proteins, which is linked to treatment resistance, but in other settings, low levels of cleaved caspases expression, opposite, are also linked to shorter survival [32]. The possible effect of therapeutic options involving the delivery of an increase or recovery in CASP3 activity, through the aid of induced apoptosis by natural compounds, or direct activation of caspases in conjunction with temozolomide and radiotherapy and predisposes the cells of glioma to death and provides new opportunities in the treatment of resistant glioblastoma [33,34].

A TNF death receptor family member (synonyms FAS/CD95/APO -1) is a transmembrane death receptor which is initiated by the Fas ligand (FasL/CD95L) and which triggers the adaptor FADD, caspase-8 activation and subsequent extrinsic apoptotic pathway that leads to fragmentation of the DNA and cell death [35,36]. In gliomas, especially GBM, tumor cells in most instances are resistant to FAS/FasL-mediated apoptosis, either due to low or heterogeneous expression of the FAS receptor in the cell surface [37]. Mutations in downstream pathways, overexpression of apoptosis inhibitors or immunosuppressive measures, such as FasL expression by glioma cells and microglia, which allows induction of counter. It has been demonstrated that FasL expression is also positively correlated with the immunosuppression of the tumor microenvironment *via* high FasL expression in gliomas and that low FAS expression on tumor cells leads to resistance to chemotherapy and radiotherapy [38]. Contrarily, in other studies, FAS stimulation is independent of apoptosis and has been demonstrated to promote cell cycle progression and proliferation of gliomas. The glioma cancer stem cells have been shown to be specifically resistant to FAS-dependent apoptosis that promotes their survival and adds to relapses [39]. Individual or combinations of therapy approaches to the FAS pathway have been shown to induce glioma cell apoptosis ex vivo and in vitro [40].

The *GPX4* gene encodes glutathione peroxidase 4, one of the major antioxidant enzymes and the major inhibitor of ferroptosis and it lowers lipid hydroperoxides to lipid alcohols using glutathione (GSH), thus preventing the accumulation of damaging lipid peroxidation end products and iron-mediated cell death in cell types with oncotic potential [41]. This is involved in preserving GBM tumor cells under ferroptosis, protecting tumor cells under therapy (temozolomide and radiotherapy) and keeping cells alive in a mesenchymal or quiescent condition of astrocyte-like cellular phenotype, and tumor progression, invasiveness and poor prognosis [42]. Glioma cells with a high expression of *GPX4* are resistant to apoptosis and ferroptosis, and down-regulation or inhibition of it causes ferroptosis, lipid ROS accumulation, impaired proliferation, migration and EMT, and interacts synergistically with conventional treatment [43]. Research has suggested that signal transmission via selective inhibition of GPX4 has been selectively sensitive to particular members of glioma subpopulations, introducing new therapeutic opportunities based on induction of ferroptosis via direct GPX4 inhibitors, to degrade it, to modulate the xCT/GSH/GPX4 axis, or to act in parallel via FSP1/CoQ10 to overcome resistance and enhance therapy response in resistant glioblastoma [44].

The ACSL4 gene (acyl-CoA synthetase long-chain family member 4) encodes a long-chain fatty acid–CoA ligase that preferentially activates polyunsaturated fatty acids (especially arachidonic and adrenic acids) into their corresponding acyl-CoA thioesters. These acyl-CoA species are subsequently incorporated into membrane phospholipids, dramatically increasing the proportion of oxidizable polyunsaturated fatty acyl chains in the lipid bilayer. This enrichment greatly sensitizes cells to lipid peroxidation, which is a central driver of ferroptosis, an iron-dependent, non-apoptotic form of regulated cell death [45,46]. ACSL4 expression is commonly decreased in gliomas as compared to normal tissue or low-grade tumors, and it adds to ferroptosis resistance and promotes tumor cell proliferation, survival and progression due to less accumulation of damaging lipid hydroperoxide [47]. It has been demonstrated that lower ACSL4 expression is associated with increased resistance to oxidative stress and drug-induced ferroptosis, and artificial overexpression or stabilization of ACSL4 induces ferroptosis, amplifying lipid peroxidation, and reducing proliferation, migration, aggressiveness and EMT of glioma cells [48]. Notably, the ACSL4 expression is higher in recurrent glioma than it is in primary glioma, which suggests that GBM relapses are more vulnerable to ferroptosis inducers [49]. Pro-ferroptosis potential is demonstrated by therapeutic approaches involving restoration or amplification of ACSL4 activity, using suppression pathway inhibitors, stimulation of expression, direct activation or combinations with GPX4/SLC7A11 inhibitors, hence creating new opportunities in the management of resistant glioblastoma by controlled delivery of ferroptosis [50].

The GCH1 gene (GTP cyclohydrolase 1) encodes the rate-limiting enzyme in the de novo biosynthesis of tetrahydrobiopterin (BH4), a crucial cofactor and endogenous antioxidant. By promoting BH4 production, GCH1 enhances cellular antioxidant defenses, facilitates lipid remodeling (including preservation of phospholipids with polyunsaturated tails), and suppresses phospholipid peroxidation. This pathway acts as a key GPX4-independent mechanism that strongly inhibits ferroptosis, an iron-dependent, non-apoptotic form of regulated cell death driven by lethal lipid peroxidation [51]. GCH1 is often overexpressed in gliomas, GBM, in brain tumor initiating cells (BTICs) and glioma stem cells (GSCs), where it facilitates preservation of their phenotype, ROS status, proliferation and growth in vivo [52]. The high expression is associated with an increase in malignancy grade, recurrence, low patient prognosis, and resistance to therapy. Experiments demonstrated that GCH1 balances GBM progression by inhibiting ferroptosis, sensitizing glioma cells to ferroptosis, amplifying the accumulation of lipid peroxides, downregulating the maintenance of BTICs/GSCs and slowing tumor growth [53]. The GCH1 mutations or genotypes are linked with a higher risk of glioma and in the context of ferroptosis, GCH1/BH4 interacts with GPX4 to protect tumor cells against ferroptosis inducers [54]. GCH1-targeted therapeutic approaches to inhibit the GCH1/BH4 axis—GCH1 knockdown, BH4 inhibitors, circLRFN5 use, natural compounds, or combinations with ferroptosis inducers—have pro-ferroptosis effects, enhance conventional therapy and demonstrate new attitudes in resistance overcoming and improving prognosis in resistant glioblastoma by triggering ferroptosis and interference with cancer stem cell maintenance [55].

SLC7A11 allows the importation of cystine and the subsequent exportation of glutamate, needed in the biosynthesis of glutathione (GSH), maintenance of redox homeostasis, and inhibition of ferroptosis through the increase in GPX4 activity and inhibiting lipid peroxide formation [56]. Overexpression of SLC7A11 is common in gliomas, specifically in GBM in tumor stem cells (GSCs/BTICs) and stem-like cells that play a role in ferroptosis resistance, survival under oxidative stress, promotion of cancer stemness properties, proliferation, invasiveness, glutamate release and worse clinical outcomes including reduced survival of high-grade patients [57]. Its high expression in SLC7A11 is associated with temozolomide (TMZ) resistance, radiotherapy and other treatments and with adaptive responses, epigenetics, lncRNAs and oncogenic pathways. Ironically, in the state of glucose starvation or large cell mass, SLC7A11 can be broken down, making it more sensitive to metabolic injury [58]. Genetic knockdown, pharmacological inhibitors, natural or regulatory pathway or combinations with ferroptosis inducers, TMZ, radiotherapy or ATM inhibitors trigger ferroptosis, lipid ROS accumulation, and reduce proliferation [59].

While 5-ALA remains the most extensively studied photosensitizer in neuro-oncology, its excitation wavelength (typically ~635 nm) limits light penetration deep into brain tissue. To overcome this, there is growing interest in repurposing indocyanine green (ICG) for PDT. The rationale for selecting ICG in this study is based on its established FDA-approved clinical safety profile, its ability to cross the disrupted blood–brain barrier in gliomas and its near-infrared (NIR) absorption peak (~800 nm). Because NIR light falls within the “biological window”, it experiences significantly less scattering and absorption by hemoglobin and water, allowing for deeper tissue penetration than visible light [60]. Although free ICG has known limitations such as rapid clearance and concentration-dependent aggregation, recent in vitro and in vivo studies have demonstrated its highly effective use as a PDT agent against glioblastoma. For example, ICG-mediated PDT has been shown to successfully induce localized cytotoxicity, trigger severe oxidative stress and even stimulate anti-tumor photoimmune responses in murine glioblastoma models [61]. Furthermore, ICG’s dual capability for both fluorescence-guided surgery (FGS) and concurrent PDT positions it as a highly promising theranostic agent for achieving complete eradication of residual glioma cells post-resection [62,63].

As genes involved in several programmed cell death (PCD) pathways are associated with prognosis in glioma and other cancer treatments [64,65] moreover glioma cells could escape PCD as those cells have a highly developed tolerance for high levels of ROS [66]. PDT consists mostly of generating an abundant amount of ROS [67]; as such, it is natural to verify the outcome of tested therapies on the expression of genes involved in PCD. In PDT studies using classical photosensitizers, a strong induction of apoptosis and/or ferroptosis at the transcriptional level is most frequently reported. The effect of PDT with indocyanine green (ICG) on the expression of genes associated with apoptosis and ferroptosis in glioma cells (T98G and U-118MG) has not been fully elucidated to date. As a result, we will test genes that relate to several PCD such as: *ACSL4*, *GPX4*, *Casp3*, *BCL2*, *BAX*, *FAS*, *SLC7A11*, *GCH1* in T98G cells after ICG-PDT and in appropriate controls: untreated cells, cells incubated with ICG alone (without irradiation), and cells irradiated without ICG to determine whether the observed cytotoxicity is associated with changes in the expression of the analyzed genes. PCR analysis will also help us verify results from sequencing analysis.

Proposed experiments could broaden the knowledge of programmed cell death pathways. Also, the knowledge about influence of photodynamic therapy on gene expression could be used in personalized medicine.

## 2. Materials and Methods

### 2.1. T98G and U-118MG Cell Cultures

To thaw the cell vial, it was placed in a water bath at 37 °C. The cells were thawed gently by agitating the vial, ensuring that the O-ring seal and vial cap were not immersed in water to reduce the risk of contamination. The thawing process took approximately 2 min. Once the vial contents were completely thawed, they were immediately removed from the water bath and disinfected by spraying the vial with 70% alcohol. From this point on, all procedures were performed under strictly aseptic conditions under a laminar flow hood. The contents of the vial were transferred to a centrifuge tube containing 9.0 mL of complete culture medium appropriate for T98G (ATCC, Manassas, VA, USA) and U-118MG (ATCC, Manassas, VA, USA) cell line: EMEM medium (EMEM, Manassas, VA, USA) supplemented with 10% fetal bovine serum (FBS, Manassas, VA, USA) and 1% antibiotic (penicillin, streptomycin, neomycin). The sample was then centrifuged at 130× *g* for 7 min. After centrifugation, the cell pellet was resuspended in the appropriate complete culture medium, and the suspension was transferred to a 75 cm^2^ culture vessel. To avoid excessive alkalinity of the medium, the vessel with the complete medium was placed in a 37 °C water bath before adding the contents of the vial to achieve a normal pH (7.0 to 7.6). The culture was incubated at 37 °C in a 5% CO_2_ atmosphere. T98G cells were passaged at appropriate rates according to ATCC recommendations, from which the cell lines were obtained.

### 2.2. Assessment of Cell Viability

After centrifugation, the cell pellet was resuspended in complete medium. A 20 μL aliquot of the cell suspension was transferred into an Eppendorf tube, and 380 μL of Muse^®^ Cell Count & Viability reagent (Luminex, Austin, TX, USA) was added. The mixture was incubated at room temperature for 5 min. Cells were then counted using the Guava^®^ MUSE^®^ Cell Analyzer (Cytek Biosciences B.V., Amsterdam, The Netherlands).

### 2.3. Experiment Design

Cell lines T98G and U-118MG were seeded in a 6-well plate (200,000 cells per well). After 24 h they were treated with ICG manufactured by Carl Roth (Karlsruhe, Germany) for PDT. One well for every type of PDT was activated with a laser beam. Then, 24 h after the laser treatment, cells were harvested and frozen at −80 °C until RNA isolation. Sample irradiation was performed using a high-intensity fiber optic illuminator with a 91 cm fiber bundle purchased from THORLABS (Newton, NJ, USA), with a power of 1.4 W at the fiber tip at maximum bulb intensity, covering a wavelength range of 400–1600 nm. All experiments were conducted a minimum of 6 times.

### 2.4. RNA Isolation

The cell pellets were lysed with lysis buffer (Qiagen, Hilden, Germany) available from the RNeasy Mini Kit (Qiagen, Hilden, Germany). The total RNA was isolated with the RNeasy Mini Kit (Qiagen, Hilden, Germany) according to the manufacturer’s protocol with DNase treatment on column. The quality and quantity were measured with a spectrophotometer (NanoPhotometer Pearl, Implen, Munich, Germany). The isolated RNA was kept at −80 °C until further analyses.

### 2.5. Reverse Transcription

A minimum of 10 ng of each probe’s RNA was transcribed into cDNA with the High-Capacity cDNA Reverse Transcription Kit (Applied Biosystems, Foster City, CA, USA) according to the manufacturer’s protocol. The reaction was done with the Mastercycler Personal Thermal Cycler (Eppendorf, Hamburg, Germany). Reaction mixture consisted of: 10 µL of diluted RNA (10 ng of RNA with nucelease-free water (EURX, Gdańsk, Poland)), 2 µL of 10× Buffer RT, 0.8 µL 25× dNTP mix (100 mM), 2 µL of 10× RT random primers, 1 µL of MultiScribe ^®^ reverse transcriptase, 1 µL of RNase inhibitor, 3.2 µL of nuclease-free water (EURX, Gdańsk, Poland). Reaction conditions were as follows: 25 °C for 10 min, 37 °C for 120 min, 85 °C for 5 min and 4 °C for ∞.

### 2.6. Gene Expression

Expression of chosen genes was assessed with RT-qPCR using TaqMan^®^ Gene Expression Assays (Assay IDs: GAPDH-Hs99999905_m1; ACSL4-Hs00244871_m1; GPX4-Hs00157812_m1; GCH1-Hs00609198_m1; SLC7A11-Hs00921938_m1; BAX-Hs00180269_m1; FAS-Hs00236330_m1; Casp3-Hs00234387_m1; BCL2-Hs00608023_m1, Applied Biosystems, Foster City, CA, USA). The reactions were done on QuantStudio 5 Real-Time PCR System (Applied Biosystems, Foster City, CA, USA) with a TaqMan^®^ Fast Advanced Master Mix for qPCR buffer (Applied Biosystems, Foster City, CA, USA). Reaction mixture consisted of: 2 µL of cDNA, 7 µL of nuclease-free water (EURX, Gdańsk, Poland), 1 µL of primer (TaqMan^®^ Gene Expression Assays), 10 µL PCR buffer (TaqMan^®^ Fast Advanced Master Mix). Reaction conditions were the same for all genes: 95 °C for 20 s, 50× (95 °C for 1 s, 60 °C for 20 s). All reactions were done in triplicate. Results were calculated with the 2^−∆∆Ct^ method using GAPDH as the reference gene and nontreated cells as the control.

### 2.7. Photosensitizer and Light Source

Indocyanine green manufactured by Carl Roth (Karlsruhe, Germany) was dissolved in sterilized water to obtain solution in the dark. Water for the preparation of stock solutions was purified using an AquaB Duo reverse osmosis system from Fresenius Medical Care, Singapore. Working ICG solutions were prepared by diluting the stock solution with serum-free medium. To each sample we added 23 μL of 4.3 × 10^−4^ M ICG and incubated for 15 min so the concentration was 4.3 μM. Irradiation was performed for 12 min 34 s at room temperature (25 °C) using a VisIR-780 picosecond-pulsed laser (PicoQuant GmbH, Berlin, Germany) operating at 780 nm. The laser output power was adjusted to ~250 mW (average power). The beam was delivered via a single-mode fiber, terminated by an adjustable achromatic collimator, and defocused/homogenized using a ground-glass diffuser (Thorlabs, Newton, NJ, USA) placed immediately above the culture plate to ensure uniform conical illumination over the entire well-plate area. The distance from the diffuser/collimator output to the cell monolayer was fixed at 20 cm. The irradiance at the cell plane was measured as approximately 40 mW/cm^2^ (using an optical power meter, averaged over the illuminated area). Fluence was calculated as irradiance × time, resulting in 30 J/cm^2^.

### 2.8. Microscopic Observations

**Figure 2:**
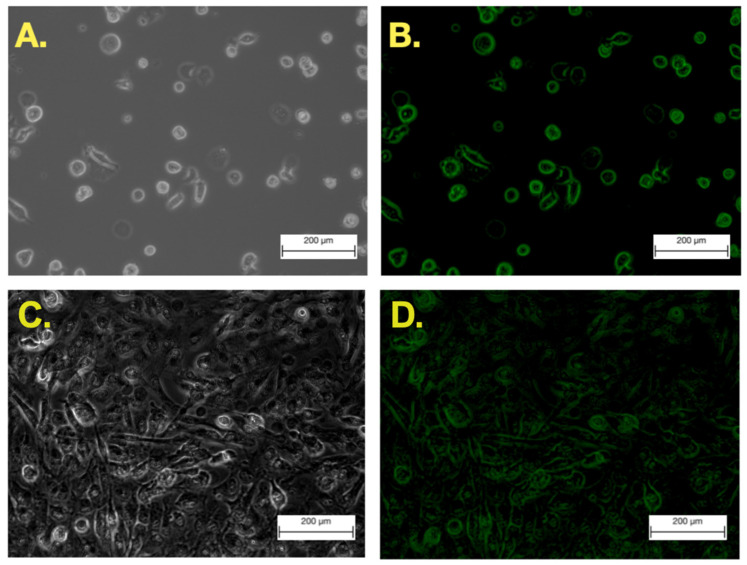
Representative microscopic images of T98G glioblastoma cells under different experimental conditions. (**A**) Untreated control cells. (**B**) Cells incubated with ICG without light exposure. (**C**) Untreated control U-118MG cells. (**D**) U-118MG cells incubated with ICG without light exposure. Images were acquired using a Zeiss Axio Imager.D2 microscope [objective 10×, scale bar = 200 μm].

Cells were observed under a microscope Zeiss Axio Imager.D2 (Oberkochen, Germany) (Figure 2).

### 2.9. Statistical Analysis

Statistical analysis was performed using GraphPad Prism version 9.0. The Shapiro–Wilk test was used to assess data normality before selecting the appropriate statistical test. Differences between the four experimental groups were assessed by one-way ANOVA followed by appropriate post hoc test when data passed the normality test. When normality assumptions were not met, the nonparametric Kruskal–Wallis test was applied. *p*-values < 0.05 were considered statistically significant. All data represent n = 6 independent biological replicates and are presented as mean ± standard deviation (SDStatistical analysis was performed using GraphPad Prism version 9.0. The Shapiro–Wilk test was used to assess data normality before selecting the appropriate statistical test. Differences between the four experimental groups were assessed by one-way ANOVA followed by appropriate post hoc test when data passed the normality test. When normality assumptions were not met, the nonparametric Kruskal–Wallis test was applied. *p*-values < 0.05 were considered statistically significant. All data represent n = 6 independent biological replicates and are presented as mean ± standard deviation (SD).

## 3. Results

The expression levels of genes associated with apoptosis (*BAX*, *BCL2*, *CASP3*, *FAS*), ferroptosis (*GPX4*, *ACSL4*, *SLC7A11*), and tetrahydrobiopterin metabolism (*GCH1*) were analyzed in T98G glioma cells subjected to four experimental conditions:

(1) Control;
(2) Control with light irradiation;
(3) Control with ICG incubation;
(4) Photodynamic therapy (ICG-PDT).

Prior to gene expression analysis, we validated that the applied PDT regimen successfully induced cell death. Using the Guava^®^ MUSE^®^ Cell Analyzer, we observed that ICG-PDT significantly decreased T98G cell viability to 67.79% ± 3.39% compared to the untreated control (86.66% ± 4.33%), light-only (88.71% ± 4.44%), and ICG-only (81.24% ± 4.06%) groups (one-way ANOVA, *p* < 0.05). In the U-118MG, ICG-PDT significantly reduced cell viability to 55.41% ± 2.19 compared to control (92.54% ± 4.01), light alone (84.51% ± 1.98), and ICG alone (82.87% ± 2.72), confirming strong phototoxicity with minimal dark toxicity. This confirms that the dosage conditions used provoked a significant cytotoxic effect, allowing us to confidently assess the corresponding transcriptional responses.

### 3.1. Gene Expression–T98G Cell Line

Each condition was tested in six independent biological replicates (n = 6). Relative expression values were calculated using the 2^−ΔΔCt^ method. Results are presented as mean ± standard deviation (Table 1). Comparison of mean expression levels between the four groups was performed using one-way ANOVA. No statistically significant differences were found in all analyzed genes (all *p* values > 0.05; range *p* = 0.347–0.905). Due to the relatively small sample size (n = 6) and the apparent heterogeneity of variance in some genes (e.g., SLC7A11, GCH1), the nonparametric Kruskal–Wallis test was additionally performed. The results of this test confirmed the lack of significant differences between groups (all *p* values > 0.05; range *p* = 0.248–0.887). While some non-significant fluctuations were observed—such as for CASP3 and GCH1 in the light-only and ICG-only groups—these were accompanied by high standard deviations (e.g., GCH1 in the control with light and ICG groups). This high variance at low baseline expression levels likely reflects technical noise rather than true biological trends. Ultimately, photodynamic therapy with ICG did not induce significant up- or down-regulation of any of the studied genes compared to the control or the other experimental conditions.

### 3.2. Gene Expression–U-118MG Cell Line

Results for U-118MG cells (n = 6) are summarized in Table 2. The results of the experiment indicate moderate changes in the expression of genes associated with apoptosis and ferroptosis under the tested conditions. The most pronounced effects were observed in the group treated with ICG-PDT, where most genes showed a tendency toward decreased expression compared to the control group. The most significant decrease was observed for the CASP3 gene (a key apoptosis effector) and BCL2 (the main anti-apoptotic gene). The expression of genes associated with ferroptosis, GPX4 and ACSL4, also decreased. The remaining genes (BAX, FAS, GCH1, SLC7A11) showed rather minor variations between groups. ICG-PDT induces a moderate decrease in the expression of both pro-apoptotic and protective genes, which may suggest the activation of mechanisms leading to cell death via apoptotic and/or ferroptotic pathways. This effect is most evident when compared to the untreated control.

### 3.3. Apoptosis-Related Genes

**Figure 3:**
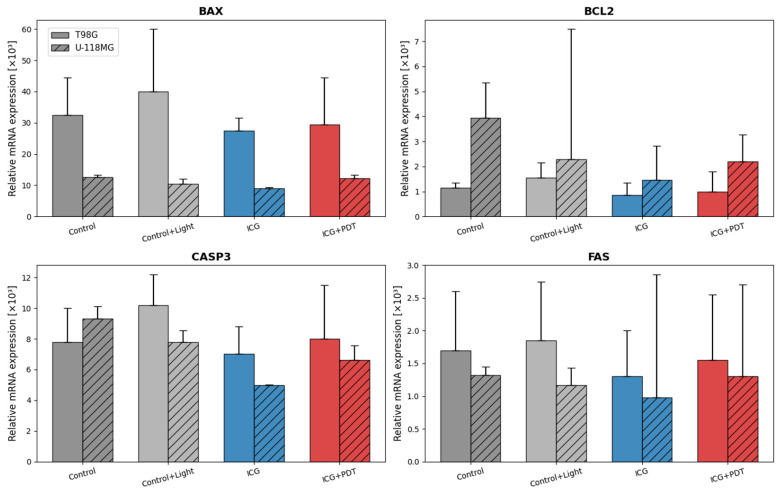
Relative mRNA expression of apoptosis-related genes (*BAX*, *BCL2*, *CASP3*, *FAS*) in T98G and U-118MG glioblastoma cells. Bars represent mean ± SD (n = 6 biological replicates) in four experimental groups: control, control treated with light (10 min broadband irradiation), ICG incubation without irradiation, and ICG-PDT (15 min ICG + 10 min broadband irradiation). Expression levels were normalized to GAPDH and calculated using the 2^−ΔΔCt^ method. No statistically significant differences were observed between groups (*p* > 0.05, Kruskal–Wallis test and ANOVA).

In the T98G line, no statistically significant changes in the expression of apoptosis-related genes (BAX, BCL2, CASP3, FAS) (Figure 3) were observed between the groups (all *p* > 0.05). The values were relatively stable, although high biological variability (high SD) was evident. In the ICG-PDT group, the expression of BAX and CASP3 was similar to that in the control group, while BCL2 and FAS did not show a clear upward or downward trend. In the U-118MG line (Figure 3), the changes were more pronounced. The most significant decrease was observed for CASP3 (*p* = 0.012 ANOVA, *p* = 0.019 Kruskal–Wallis)—the lowest expression was recorded in the Control with ICG group (4.98 × 10^−3^), while in the ICG-PDT group it was 6.60 × 10^−3^ (compared to 9.32 × 10^−3^ in the control). BCL2 also showed a downward trend in the ICG and ICG-PDT groups. BAX and FAS showed no significant differences. Overall, in U-118MG, ICG-PDT caused a moderate reduction in both pro-apoptotic (CASP3) and anti-apoptotic (BCL2) genes, which may suggest the activation of apoptotic pathways, but at the same time a weakening of protective mechanisms.

### 3.4. Ferroptosis-Related Genes

**Figure 4:**
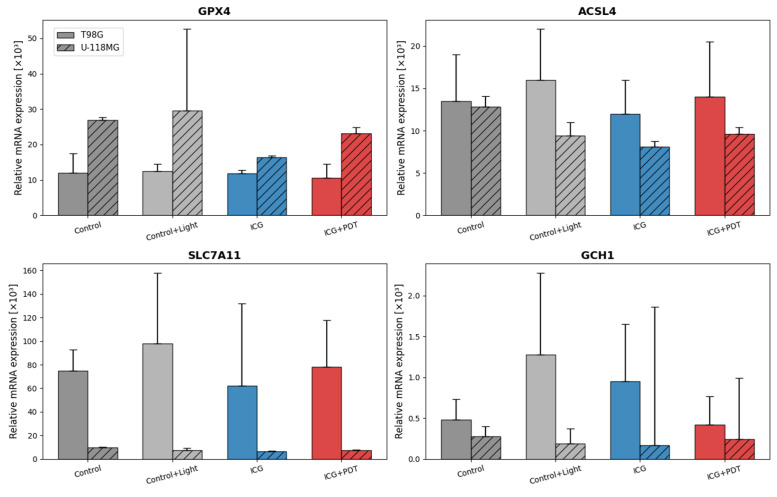
Relative mRNA expression of ferroptosis-related genes (GPX4, ACSL4, SLC7A11, GCH1) in T98G and U-118MG glioblastoma cells. Bars represent mean ± SD (n = 6) in control, control with light, ICG-only, and ICG-PDT groups. Data were normalized to GAPDH and analyzed using the 2^−ΔΔCt^ method. No significant changes in expression were detected across groups (all *p* > 0.05). The largest non-significant trend was a mild decrease in GPX4 in the ICG-PDT group (fold change 0.87).

In the T98G line (Figure 4), no statistically significant differences were observed in the GPX4, ACSL4, GCH1, and SLC7A11 genes (all *p* > 0.05). SLC7A11 expression was relatively high in all groups, while GPX4 and ACSL4 remained stable. No clear effect of ICG-PDT on ferroptosis was observed in this model. More pronounced effects were observed in the U-118MG line (Figure 4). ACSL4 (pro-ferroptotic) showed a significant decrease (*p* = 0.031 ANOVA, *p* = 0.038 Kruskal–Wallis) in all experimental groups, most pronounced in the Control with ICG group. GPX4 (a key anti-ferroptotic factor) also showed a downward trend (*p* = 0.085), particularly in the ICG group (16.36 × 10^−3^ vs. 26.86 × 10^−3^ in the control). SLC7A11 and GCH1 did not show significant changes, although the trend was rather downward in the treated groups.

## 4. Discussion

Although no statistically significant changes in mRNA expression were observed, these negative findings are important because they contrast with most studies using other photosensitizers (e.g., 5-ALA-PDT strongly upregulates BAX/BCL2 ratio and CASP3 while downregulating GPX4). This suggests that ICG-PDT induces cytotoxicity in T98G cells primarily through post-transcriptional mechanisms or direct organelle damage by singlet oxygen rather than transcriptional reprogramming of classical apoptosis/ferroptosis pathways. Deregulated apoptosis and ferroptosis pathways are a characteristic feature of gliomas, particularly GBM, contributing to their resistance to standard treatments. Inhibition of apoptosis is often observed in GBM, leading to evasion of classical cell death [68,69]. At the same time, GBM cells exhibit dysregulation of ferroptosis, including increased antioxidant activity and altered iron and lipid metabolism, which allows them to resist the induction of lipid peroxidation and ferroptotic death [43,53]. These mechanisms collectively promote resistance to chemotherapy, radiotherapy, and new targeted therapies, worsening patient prognosis. The *BAX* (proapoptotic) and *BCL2* (antiapoptotic) genes control the mitochondrial apoptotic pathway; *CASP3* is an executor, *FAS (CD95)* is an activator of the extrinsic (receptor) pathway; *GPX4* and *SLC7A11* (xCT) inhibit ferroptosis, *ACSL4* promotes it, and *GCH1* suppresses ferroptosis through the BH4 axis, being a GPX4-independent suppressor [70,71]. Comparison of purely published information provided at PubMed can prove the alterations in the expression of these markers, which are caused by the therapy. Low apoptotic rate and high levels of ferroptosis suppressors prevail in untreated gliomas [47,57,72]. In T98G, TMZ mostly causes mitochondrial apoptosis: BAX increase, BCL2 decrease (BAX/BCL2 ratio increase), and CASP3/7 activation (Western blot, MTT; concentrations 1000–2000 μM) [73]. In part, it regulates ferroptosis, upregulation of DMT1, accompanied by an offsetting increase in GPX4/SLC7A11 in resistance [74]. Relapses are repressed by SLC7A11 (ATM/p53-dependent) in combination with a reduction in GPX4 and an increase in ACSL4 [49]. GCH1 (PRRX2/GCH1/BH4 axis) offers defense to GSCs; its inhibition increases the impact of RT [71]. PDT is characterized by the greatest range of inciting cell death, given the fact that the local formation of ROS (mainly singlet oxygen and superoxide) was conducted in a highly diverse range of papers published in PubMed. The data are obtained in the works on U87MG, U138, U251, T98G cell lines, patient-derived GBM, in vivo models (C6, GL261), and other photosensitizers (5-ALA, Photogem^®^, talaporfin sodium, hypericin, TiO2/ZnO nanoparticles). In relation to apoptosis, the findings are always proapoptotic. As shown in the U87MG cell line by Karmakar et al., the survival factors (NFκB, BIRC-3, BCL2) are suppressed, the BAX /BCL2 ratio is elevated, cytochrome c and AIF are released, and the calpain and caspase cascade (caspase-9 and CASP3) are stimulated by 5-ALA-PDT, which is confirmed by Western blot and enzyme activity [75]. The authors emphasized that the mitochondrial apoptotic pathway is the dominant mechanism of action of 5-ALA-PDT in U87MG cells and that the suppression of survival factors (NFκB, BIRC-3, BCL2) is a key therapeutic target. Tirapelli et al. examined PDT using Photogem^®^ (25 J/cm^2^, 630 nm, 5 μg/mL). Only in U87 and U138, a significant increase in CASP3 mRNA (real-time qPCR, *p* < 0.05) and a reduction in viability (Trypan blue) were found, which shows that the response to p53 mutations was heterogeneous [76]. Crucially, the unique cytogenetic background of these cell lines profoundly influences the PDT response pathway. Unlike the U87 cell line (which is p53 wild-type and highly sensitive to classical apoptosis), T98G cells are characterized by a mutant p53 and PTEN wild-type status, contributing to their highly invasive and therapy-resistant phenotype. Because p53 is a master transcriptional regulator of apoptosis and ferroptosis, its mutated status in T98G strongly raises the cell death threshold and blunts normal transcriptional responses to oxidative stress. This unique genetic background provides a compelling explanation for why our ICG-PDT regimen successfully induced cytotoxicity without the classical mRNA upregulation of apoptosis or ferroptosis markers, underscoring the necessity of evaluating PDT across diverse cytogenetic profiles. When combined with BCL2/BCL-xL inhibitor (ABT-263/navitoclax) the apoptosis is synergistically increased, caspase-dependently. CASP9 cleavage increased, CASP3 cleavage increased, Noxa/Mcl-1 ratio increased (independent of Usp9X) facilitating BAX/BAK release [77]. The combination of PDT with navitoclax produces a clear caspase-dependent synergy by modulating the Noxa/Mcl-1 ratio, which opens the way to combination strategies. Li et al. noted that CASP3 apoptotic protein (Western blot) was activated, mitochondrial damage and anaerobic glycolysis were reprogrammed in PDT + TMZ, and the synergy enhanced apoptosis in comparison with monotherapy [78]. The authors conclude that PDT + TMZ synergistically enhances apoptosis by reprogramming glucose metabolism and mitochondrial damage, which warrants further clinical studies. The results of Miki et al. with materials containing talaporfin sodium + TMZ showed an upsurge in CASP3 activity and fragmentation of DNA (flow cytometry) [79]. Further improvements by Pevna et al. (hypericin-PDT in U87MG) raised CASP3 and autophagy/apoptosis [80]. Teixeira et al. [81] showed increased BAX, decreased BCL2, CASP3 activation, and inhibition of GPX4 activity. In summary, most preclinical studies indicate a central role for mitochondrial apoptosis in the mechanism of PDT in glioblastoma and a clear potential for synergy with TMZ and BCL-2/xL inhibitors. However, heterogeneity of response and the need to optimize PDT parameters and photosensitizer selection remain major challenges before moving to phase II/III clinical trials. The FAS (death receptor) pathway is less commonly directly stimulated in glioma PDT. There is an intrinsic pathway that dominates, but ROS can also indirectly regulate the death receptors (only some cases have been described, usually in other cancer models). Permitting the context of ferroptosis, the PDT produced is very high levels of ROS, which provoke lipid peroxidation and cause ferroptosis-like death. In the present study, however, ICG-mediated PDT applied to T98G glioblastoma cells did not induce any statistically significant gene changes in the examined panel of apoptosis- and ferroptosis-related genes 24 h post-treatment. Specifically, no significant alterations were observed for *BAX*, *BCL2*, *CASP3* or *FAS* (apoptosis) nor for *GPX4*, *ACSL4*, *SLC7A11* or *GCH1* (ferroptosis) (Figure 3 and Figure 4). These findings contrast with most reports using other photosensitizers and indicate that, under the applied experimental conditions, ICG-PDT cytotoxicity in T98G cells at a given concentration of ICG is not driven by reprogramming of the classical apoptotic or ferroptotic pathways. Hsia et al. [13] refer to the fact that PDT triggers ferroptosis in gliomas. The synergy between PDT and ferroptosis is demonstrated through reviews [6] because both interfere with the ROS and GSH metabolism. ACSL4 and GCH1 data under pure PDT have little literature (primarily indirect: ROS favors ACSL4 activation and suppresses protective GCH1/BH4) [82,83] but a higher level of ferroptosis susceptibility was found in model nanoparticles [84]. In contrast, the U-118MG cell line showed greater sensitivity at the transcriptional level. ICG-PDT induced a statistically significant decrease in CASP3 and ACSL4 expression, along with downward trends in BCL2 and GPX4. This pattern suggests that in U-118MG cells, ICG-PDT triggers moderate activation of apoptotic and ferroptotic pathways, accompanied by a weakening of cellular protective mechanisms (reduced anti-apoptotic BCL2 and anti-ferroptotic GPX4). Such a dual effect may indicate simultaneous engagement of both cell death routes, which is consistent with the strong ROS-generating nature of PDT. The difference in response between the two cell lines is noteworthy. While T98G cells exhibited primarily post-transcriptional cytotoxicity without significant mRNA changes, U-118MG cells displayed clearer transcriptional reprogramming. This heterogeneity aligns with previously reported variable responses of glioblastoma cell lines to PDT, which often depend on genetic background, p53 status, and basal antioxidant capacity. PDT is characterized by the co-existence of these two pathways because local ROS in the mitochondria/lysosomes result in both pathways being activated simultaneously compared to TMZ (apoptosis-dominant) or RT [85,86]. Therapies that target ferroptosis in GPX4 (down), ACSL4 (up), SLC7A11 (down) and GCH1 (down through circLRFN5) are maximized [71,87]. Comparative analysis of therapies shows: TMZ and PDT most robustly activate BAX/ decrease BCL2/ upsurge CASP3 (PDT also co-operates with BCL2 inhibitors) [78,88]; RT and PDT most robustly lower GPX4/SLC7A11 by ROS [53,89]; ACSL4 increases in relapses following RT + TMZ and PDT -nanoparticles [48]; GCH1 is a stable suppressor, and its downregulation amplifies the action of PDT/RT [71,90]; FAS is minimally altered. The resistance seen using GBM is due to the compensatory upsurge in the levels of BCL2, GPX4, SLC7A11 and GCH1; therefore, the combination of PDT + TMZ (elevated TMZ levels in tissue, apoptosis, inhibition of migration) is more efficient than monotherapies in GBM [74,79,80]. While TMZ and RT consistently alter the BAX/BCL2 ratio and GPX4/SLC7A11 levels, and PDT with 5-ALA, hypericin or nanoparticle photosensitizers robustly activate both apoptosis and ferroptosis at the transcriptional level, ICG-PDT in T98G cells failed to elicit any significant mRNA changes in the eight-gene panel. This lack of gene expression change, despite documented cytotoxicity of the treatment, points to post-transcriptional mechanisms or direct organelle damage. As demonstrated in other PDT models, photochemically generated singlet oxygen (^1^O_2_) can instantaneously peroxidize membrane lipids and oxidize mitochondrial proteins. This leads to a rapid loss of mitochondrial membrane potential and immediate caspase activation without requiring de novo mRNA synthesis [67,82,91,92]. Alternatively, the cells may be undergoing non-canonical cell death routes such as necroptosis, which are heavily protein-driven [93,94]. While PDT with many photosensitizers induces robust transcriptional changes leading to apoptosis and ferroptosis, ICG-PDT in our model relies predominantly on post-transcriptional mechanisms, highlighting photosensitizer-specific differences. Recent literature emphasizes that the primary determinant of the cell death pathway and overall outcome in PDT is the specific subcellular localization and characteristics of the chosen photosensitizer [95]. Although most PDT studies using other photosensitizers demonstrate a comprehensive cell death phenotype involving both apoptosis (↑BAX/BCL2 ratio, ↑CASP3) and ferroptosis (↓GPX4/SLC7A11, ↑ACSL4), the present ICG-PDT experiment in T98G glioblastoma cells revealed no statistically significant gene changes in this eight-gene panel 24 h after treatment. The observed cytotoxicity may therefore result from rapid post-transcriptional and post-translational events or non-canonical pathways. Indocyanine green PDT under the experimental conditions used does not induce significant gene expression in genes regulating apoptosis and ferroptosis in T98G glioma cells. In U-118MG cells, however, ICG-PDT produced measurable downregulation of key genes (CASP3, ACSL4, trends in BCL2 and GPX4), indicating partial engagement of apoptotic and ferroptotic pathways at the transcriptional level. The previously observed cytotoxicity of PDT may therefore result from different mechanisms, for example, protein activation, post-translational modifications, direct organelle damage by singlet oxygen or other pathways of cell death. The high reproducibility of the method and the lack of effect in the ICG group without light confirm the specificity of the lack of effect on mRNA levels. The results indicate that PDT with ICG does not transcriptionally trigger or inhibit ferroptosis in the model studied. These findings highlight the ICG nature in PDT molecular responses and suggest that the tested gene panel may serve as a biomarker panel. Future clinical and preclinical studies directly comparing ICG-PDT with TMZ and RT at both mRNA and protein levels are warranted to optimize adjuvant PDT strategies in glioblastoma. The present study has several limitations that must be addressed in future research. First, as this was primarily a transcriptomic screen, cytotoxicity was validated via cell viability counting (Guava MUSE analysis). While this confirmed the biological efficacy of the ICG-PDT regimen, specific phenotypic validations such as LDH release assays, Annexin V/PI flow cytometry, or Western blotting for cleaved caspases and lipid peroxidation markers are needed to definitively characterize the execution mechanism. Second, our transcriptomic analysis was limited to a single time point (24 h post-treatment). It is highly plausible that ICG-PDT induces an early, transient wave of transcriptional regulation (e.g., at 2, 4, or 12 h) that resolves by 24 h. Future studies must employ a time-course mRNA and protein analysis across a broader panel of glioma cell lines (e.g., U87MG, U251) to fully elucidate the complex, likely multifactorial cell death pathways triggered by ICG-PDT.

## 5. Conclusions

Under the experimental conditions employed in this study, indocyanine green-mediated photodynamic therapy (ICG-PDT) induced cell-line-dependent effects on the mRNA expression of major regulators of apoptosis (BAX, BCL2, CASP3, FAS) and ferroptosis (GPX4, ACSL4, SLC7A11, GCH1) at the 24 h time point. In T98G glioblastoma cells, ICG-PDT did not induce any statistically significant changes in the expression of the analyzed genes. Only minor, non-significant fluctuations were observed, including a slight reduction in GPX4 in the PDT group. The mRNA levels of BAX and BCL2 remained stable, with no significant alteration in their relative ratio. These findings indicate that the cytotoxic effect of ICG-PDT in the T98G model is not driven by substantial transcriptional reprogramming of the classical apoptotic or ferroptotic pathways. In contrast, the U-118MG cell line showed greater transcriptional sensitivity. ICG-PDT induced statistically significant decreases in CASP3 (*p* = 0.012) and ACSL4 (*p* = 0.031) expression, accompanied by downward trends in BCL2 and GPX4. This pattern suggests moderate engagement of both apoptotic and ferroptotic pathways together with a partial weakening of cellular protective mechanisms in U-118MG cells. Overall, the observed cytotoxicity of ICG-PDT is most likely mediated by rapid post-transcriptional events, direct photochemical damage to organelles (mitochondria, membranes) by singlet oxygen, protein oxidation, or activation of non-canonical cell death routes, with the contribution of transcriptional changes being strongly cell-line dependent. The high reproducibility of the results and the absence of unspecific effects in the ICG-only (dark toxicity) group support the specificity of these findings. The present study is limited to mRNA analysis at a single time point (24 h) in two cell lines. Therefore, future investigations should focus on: (I) functional assays to definitively confirm the execution of apoptosis and/or ferroptosis at the cellular level, (II) protein-level expression and activation (Western blot, activity assays), (III) earlier time points (e.g., 2–6 h post-irradiation) to capture possible transient transcriptional responses, and (IV) a broader panel of glioblastoma models, including patient-derived primary cultures. Only after the precise mode of cell death induced by ICG-PDT is firmly established in different biological contexts should the rational design of combination treatments such as those utilizing ferroptosis inducers or BCL-2 family modulators be considered to enhance efficacy in resistant glioblastoma cells.

## Figures and Tables

**Table 1:** Relative mRNA expression of the studied genes in T98G cells (mean ± SD, n = 6). Expression values are presented as ×10^−3^ (mean ± SD).

Gene	Control	Control with Light	Control with ICG	ICG-PDT	*p* (ANOVA)	*p* (Kruskal–Wallis)
BAX	33.18 ± 10.59	40.07 ± 20.03	26.61 ± 5.70	29.40 ± 15.74	0.684	0.536
BCL2	1.11 ± 0.13	1.54 ± 1.22	0.85 ± 0.47	0.96 ± 0.82	0.717	0.789
CASP3	7.66 ± 2.36	10.44 ± 1.88	6.84 ± 1.61	7.90 ± 3.32	0.347	0.248
GPX4	11.80 ± 5.86	12.32 ± 1.25	11.73 ± 0.91	10.23 ± 4.03	0.905	0.715
ACSL4	13.59 ± 4.90	16.16 ± 7.06	11.88 ± 4.16	13.90 ± 7.03	0.850	0.887
FAS	1.68 ± 0.95	1.82 ± 0.91	1.29 ± 0.76	1.52 ± 0.99	0.883	0.679
GCH1	0.42 ± 0.30	1.25 ± 1.07	0.94 ± 0.72	0.39 ± 0.40	0.393	0.306
SLC7A11	76.27 ± 17.17	101.60 ± 59.77	62.18 ± 71.59	78.29 ± 36.86	0.818	0.789

**Table 2:** Relative mRNA expression of the studied genes in U-118MG cells (mean ± SD, n = 6). Expression values are presented as ×10^−3^ (mean ± SD).

Gene	Control	Control with Light	Control with ICG	ICG-PDT	*p* (ANOVA)	*p* (Kruskal–Wallis)
BAX	12.54 ± 0.70	10.52 ± 1.46	9.04 ± 0.38	12.17 ± 1.21	0.142	0.148
BCL2	3.94 ± 1.40	2.28 ± 5.21	1.45 ± 1.38	2.19 ± 1.09	0.478	0.521
CASP3	9.32 ± 0.78	7.79 ± 0.75	4.98 ± 0.04	6.60 ± 0.98	0.012	0.019
GPX4	26.86 ± 0.80	29.50 ± 23.12	16.36 ± 0.43	23.17 ± 1.77	0.085	0.067
ACSL4	12.81 ± 1.25	9.41 ± 1.57	8.07 ± 0.71	9.62 ± 0.80	0.031	0.038
FAS	1.32 ± 0.13	1.17 ± 0.26	0.98 ± 1.88	1.30 ± 1.40	0.912	0.876
GCH1	0.28 ± 0.12	0.19 ± 0.18	0.17 ± 1.69	0.24 ± 0.75	0.845	0.791
SLC7A11	9.63 ± 0.73	7.31 ± 2.00	6.42 ± 0.72	7.33 ± 0.32	0.214	0.237

## Data Availability

The original contributions presented in this study are included in the article. Further inquiries can be directed to the corresponding author.
